# Diabetes is a risk factor for the progression and prognosis of COVID‐19

**DOI:** 10.1002/dmrr.3319

**Published:** 2020-04-07

**Authors:** Weina Guo, Mingyue Li, Yalan Dong, Haifeng Zhou, Zili Zhang, Chunxia Tian, Renjie Qin, Haijun Wang, Yin Shen, Keye Du, Lei Zhao, Heng Fan, Shanshan Luo, Desheng Hu

**Affiliations:** ^1^ Department of Integrated Traditional Chinese and Western Medicine, Union Hospital, Tongji Medical College Huazhong University of Science and Technology Wuhan China; ^2^ Department of Neurosurgery, Union Hospital, Tongji Medical College Huazhong University of Science and Technology Wuhan China; ^3^ Department of Infectious Disease, Union Hospital, Tongji Medical College Huazhong University of Science and Technology Wuhan China; ^4^ Institute of Hematology, Union Hospital, Tongji Medical College Huazhong University of Science and Technology Wuhan China

**Keywords:** COVID‐19, diabetes, prognosis

## Abstract

**Backgound:**

To figure out whether diabetes is a risk factor influencing the progression and prognosis of 2019 novel coronavirus disease (COVID‐19).

**Methods:**

A total of 174 consecutive patients confirmed with COVID‐19 were studied. Demographic data, medical history, symptoms and signs, laboratory findings, chest computed tomography (CT) as well the treatment measures were collected and analysed.

**Results:**

We found that COVID‐19 patients without other comorbidities but with diabetes (n = 24) were at higher risk of severe pneumonia, release of tissue injury‐related enzymes, excessive uncontrolled inflammation responses and hypercoagulable state associated with dysregulation of glucose metabolism. Furthermore, serum levels of inflammation‐related biomarkers such as IL‐6, C‐reactive protein, serum ferritin and coagulation index, D‐dimer, were significantly higher (*P* < .01) in diabetic patients compared with those without, suggesting that patients with diabetes are more susceptible to an inflammatory storm eventually leading to rapid deterioration of COVID‐19.

**Conclusions:**

Our data support the notion that diabetes should be considered as a risk factor for a rapid progression and bad prognosis of COVID‐19. More intensive attention should be paid to patients with diabetes, in case of rapid deterioration.

## 1 INTRODUCTION

Since the end of 2019, a newly identified illness termed COVID‐19 has spread rapidly through China and the rest of the world. A novel beta‐coronavirus, known as the severe acute respiratory syndrome corona virus 2 (SARS‐CoV‐2), was identified as the COVID‐19 pathogen, which triggered severe pneumonia and acute, even lethal, lung failure.^1^ By 2 March 2020, the virus had caused 80 303 confirmed cases and 2948 deaths in China and cases have been reported in 45 countries, including the United States, Korea, Japan and Italy. In the existing cases, some patients with SARS‐CoV‐2 pneumonia developed acute respiratory distress syndrome (ARDS), and a part of them worsened in a short period of time and died of multiple organ failure.^1^

Angiotensin‐converting enzyme 2 (ACE2) is the surface receptor for SARS coronavirus (SARS‐CoV), directly interacting with the spike glycoprotein (S protein).^2^ A recent study suggests that the affinity between ACE2 and the receptor‐binding domain (RBD) of SARS‐CoV‐2 is 10 to 20 times higher than that with the RBD of SARS‐CoV, indicating that ACE2 might also be the receptor for SARS‐CoV‐2.^3^ ACE2 was reported to be widely expressed in various organ systems including the cardiovascular system, kidneys, lungs and brain,^4^, ^5^ which might provide an explanation for why some COVID‐19 patients died of multiple organ failure.

Diabetes mellitus is one of the leading causes of morbidity worldwide and is anticipated to rise substantially over the next decades.^6^ Several investigations have demonstrated a higher susceptibility to some infectious diseases in diabetic people, like *Staphylococcus aureus* and *Mycobacterium tuberculosis*,^7^, ^8^, ^9^ probably owing to the dysregulated immune system.^10^ It has reported that plasma glucose levels and diabetes are independent predictors for mortality and morbidity in patients with SARS.^11^ A retrospective study in Wuhan, China revealed that of the 41 COVID‐19 patients, 32% of them had underlying diseases, and among which 20% was diabetes.^12^ Therefore, these diabetic patients might be at increased risk of COVID‐19 and have a poorer prognosis.

To figure out whether diabetes is a risk factor to influence the progression and prognosis of COVID‐19, 174 COVID‐19 patients who were admitted to Wuhan Union Hospital from 10 February 2020 to 29 February 2020 were included in this study according to the inclusion criteria. Their basic information, laboratory examinations, chest computed tomography (CT) scans as well the treatment measures were collected and analysed. We found that, as a common underlying disease in COVID‐19 patients, diabetes is associated with worse prognosis.

## 2 MATERIALS AND METHODS

This was a retrospective study of 174 patients with SARS‐Cov‐2 infection who were admitted to Wuhan Union hospital from 10 February 2020 to 29 February 2020. The enrolled patients in this study are all diagnosed with COVID‐19 according to WHO interim guidance. Wuhan Union Hospital is a designated hospital for treating COVID‐19. In the first part, we divided all patients into two groups based on whether they had diabetes. In the second part, we excluded patients with comorbidities other than diabetes to avoid the impact of other comorbidities, and then the patients with diabetes and patients without diabetes were separated into two groups. This case series was approved by the Institutional Ethics Board of Wuhan Union Hospital of Tongji Medical College, Huazhong University of Science and Technology. Written informed consent was waived by the Ethics Commission of the designated hospital for the emerging infectious diseases.

We extracted demographic data, medical history, exposure history, symptoms and signs, laboratory findings, chest CT scans, and the treatment measures from electronic medical records. The date of disease onset was defined as the day when the first symptom showed up. Clinical outcomes were followed up to 3 March 2020. All data were analysed by the research team and double checked by two physicians (Y.S. and L.Z.).

Laboratory validation of SARS‐CoV‐2 was performed at Wuhan Union Hospital. Throat‐swab specimens that obtained from the upper respiratory tract of patients at admission were stored in viral‐transport medium. Total RNA was extracted within 2 hours using the respiratory sample RNA isolation kit (Zhongzhi, Wuhan, China). SARS‐CoV‐2 was examined by RT‐PCR as described previously.^12^

All COVID‐19 patients met the following criteria: (a) Epidemiology history, (b) Fever or other respiratory symptoms, (c) Typical CT image abnormities of viral pneumonia, and (d) Positive result of RT‐PCR for SARS‐CoV‐2 RNA. Patients were divided into the diabetes and non‐diabetes group according to their medical history. Furthermore, CT imaging scores were used to quantify the pathological changes of COVID‐19 patients. The values were obtained by two physicians, who were blinded to the patients' clinical data, using an introduced scoring system described as below.

**Table:** 

No.	Performance	Score
(1)	Unbilateral patchy shadows or ground‐glass opacity	5
(2)	Bilateral patchy shadows or ground‐glass opacity	7
(3)	Diffuse changes for (1) or (2)	2
(4)	Unbilateral solid shadow, strip shadow	2
(5)	Bilateral solid shadow, strip shadow	4
(6)	Unbilateral pleural effusion	2
(7)	Bilateral pleural effusion	4
(8)	Increased or enlarge mediastinal lymph nodes	1

Categorical variables were expressed as frequency rates and percentages (%), and continuous variables were expressed as mean (SD) if they are normally distributed or median (IQR) if not. Categorical variables between groups were compared using the *χ*2 test or Fisher exact test, and continuous variables were analysed using Student's *t* test or Mann‐Whitney *U* test as appropriate. All statistical analyses were performed using the SPSS 13.0 software. ^a^ *P*‐value <.05 was considered statistically significant.

## 3 RESULTS

Of 174 hospitalized patients with COVID‐19, the median age was 59 years (interquartile range, 49‐67) and 76 (43.7%) were men. For the all patients the most common symptoms were fever (78.2%), chill (68.4%), cough (32.2%), fatigue (27%), chest tightness (25.9%), shortness of breath (24.1%) and myalgia (20.7%), whereas nausea (9.8%), headache (6.9%), pharyngalgia (5.2%) and chest pain (8.6%) were relatively rare. The most common of underlying comorbidities were chronic diseases, such as hypertension (24.7%) and diabetes (21.2%). Compared with patients without diabetes, patients with diabetes had more cardiovascular disease (32.4% vs 14.6%) and less fever (59.5% vs 83.2%), but had no significant differences in gender and age, as well as mortality (Table 1).

**TABLE 1:** Demographics and baseline characteristics of patients infected with SARS‐CoV‐2

	No. (%) Total (n = 174)	Non‐diabetes (n = 137)	Diabetes (n = 37)	*P*‐value^a^
Age, median (IQR), y	59 (49‐67)	58 (47‐66)	61 (55‐69)	.054
Gender				
Male	76 (43.7)	56 (40.9)	20 (54.1)	.152
Female	98 (56.3)	81 (59.1)	17 (45.9)	
Comorbidities				
Hypertension	43 (24.7)	33 (24.1)	10 (27)	.713
Cardiovascular disease	32 (18.4)	20 (14.6)	12 (32.4)	.013
Malignancy	17 (4.6)	16 (11.7)	1 (2.7)	.187
Pulmonary disease	14(9.7)	12 (8.7)	2 (5.4)	.745
Cerebrovascular disease	13 (7.5)	12 (8.7)	1 (2.7)	.373
Chronic kidney disease	13 (7.5)	12 (8.7)	1 (2.7)	.373
Chronic liver disease	8 (4.6)	8 (5.8)	0	.288
Immunodeficiency	4 (2.3)	4 (2.9)	0	.294
Hepatitis B infection	2 (1.1)	2 (1.5)	0	.461
Signs and symptoms				
Fever	136 (78.2)	114 (83.2)	22 (59.5)	.002
Highest temperature, °C				
<37.3	38 (21.8)	23 (16.8)	15 (40.5)	.002
37.3 to 38.0	36 (20.7)	28 (20.4)	8 (21.6)	.875
38.1 to 39.0	73 (42)	62 (45.3)	11 (29.7)	.089
>39.0	27 (15.5)	24 (17.5)	3 (8.1)	.161
Fatigue	47 (27)	36 (26.3)	11 (29.7)	.675
Chill	119 (68.4)	98 (71.5)	21 (56.8)	.086
Cough	56 (32.2)	48 (35)	8 (21.6)	.121
Pharyngalgia	9 (5.2)	8 (5.8)	1 (2.7)	.729
Dizziness	23 (13.2)	17 (12.4)	6 (16.2)	.739
Headache	12 (6.9)	10 (7.3)	2 (5.4)	.970
Chest tightness	45 (25.9)	40 (29.2)	5 (13.5)	.053
Chest pain	15 (8.6)	14 (10.2)	1 (2.7)	.265
Shortness of breath	42 (24.1)	37 (27)	5 (13.5)	.089
Myalgia	36 (20.7)	30 (21.9)	6 (16.2)	.449
Nausea and vomiting	17 (9.8)	12 (8.8)	5 (13.5)	.581
Diarrhoea	21 (12.1)	18 (13.1)	3 (8.1)	.583
Mortality	9 (5.2)	5 (3.6)	4 (10.8)	.185

a *P* values indicate differences between diabetes and non‐diabetes patients. *P* < .05 was considered statistically significant.

Of all patients, there were many typically abnormal laboratory test results (Table 2), including enzymes like α‐hydroxybutyrate dehydrogenase (HBDH; 190 [IQR, 146‐263]), alanine aminotransferase (ALT; 26 [IQR,21‐37]), lactic dehydrogenase (LDH; 248 [IQR, 188‐362]) and inflammation‐related markers, such as C‐reactive protein (CRP; 17.7 [IQR, 7.34‐51.8]), serum ferritin (375.9 [IQR, 169.5‐746.9]), erythrocyte sedimentation rate (ESR; 28 [IQR, 13‐59]), IL‐6 (11.75 [IQR, 5.1‐28.2]), as well as coagulation parameters, such as D‐dimer (0.67 [IQR, 0.3‐1.4]) and fibrinogen (FIB; 4.78 [IQR, 3.28‐5.8]). In addition, the absolute counts of lymphocytes (0.96 [IQR, 0.7‐1.3]) and neutrophils (2.7 [IQR, 1.8‐4.6]) also show abnormal change.

**TABLE 2:** Comparison of laboratory parameters between diabetic and non‐diabetic COVID‐19 patients

		Median (IQR)			
	Normal range	Total (n = 174)	Non‐diabetes (n = 137)	Diabetes (n = 37)	*P*‐value^a^
HBDH (U/L)	72 to 182	190 (146–263)	190 (143.5‐251.5)	210 (177‐480)	.13
ALT (U/L)	5 to 35	26 (21‐37)	25 (17‐42)	28 (21‐34)	.2
LDH (U/L)	109 to 245	248 (188‐362)	241 (187‐372.3)	252 (174.5‐292.5)	.76
GGT (U/L)	11 to 50	25 (14‐51.3)	24 (14‐45)	32 (17.5‐52)	.19
Lymphocytes (×10^9^/L)	1.1 to 3.2	0.96 (0.7‐1.3)	0.97 (0.74‐1.3)	0.86 (0.5‐1.3)	.04
Neutrophils (×10^9^/L)	1.8 to 6.3	2.7 (1.8‐4.6)	2.5 (1.6‐3.7)	4.1 (2.8‐6.9)	<.01
Red blood cells (×10^12^/L)	3.8 to 5.1	4.14 (3.8‐4.4)	4.17 (3.8‐4.5)	3.9 (3.5‐4.2)	<.01
Haemoglobin (g/dL)	115 to 150	124 (115‐135)	127 (117‐136)	117 (105‐123.5)	<.01
C‐reactive protein (mg/L)	<8	17.7 (7.34‐51.8)	16.3 (7.17‐43.9)	32.8 (11.3‐93)	.06
Serum ferritin (ng/ml)	21.8 to 275	375.9 (169.5‐746.9)	372.6 (185.8‐685.8)	594.4 (164‐1146.2)	.15
ESR (mm/h)	<15	28 (13‐59)	23 (10‐49)	67 (47.5‐81)	<.01
IL‐6 (pg/ml)	0.1 to 2.9	11.75 (5.1‐28.2)	11.16 (4.5‐25)	18.3 (7.3‐37.6)	.07
D‐dimer (μg/L)	<0.5	0.67 (0.3‐1.4)	0.54 (0.25‐1.1)	1.15 (0.83‐2.11)	<.01
FIB (g/L)	2.0 to 4.0	4.78 (3.8‐5.8)	4.58 (3.7‐5.6)	5.1 (4.6‐6.3)	.27

Abbreviations: ALT, alanine aminotransferase; COVID‐19，coronavirus disease 2019; ESR, erythrocyte sedimentation rate; FIB, fibrinogen; GGT, γ‐glutamyltransferase; HBDH, α‐hydroxybutyrate dehydrogenase; IQR, interquartile range; LDH, lactic dehydrogenase.

a *P* values indicate differences between diabetes and non‐diabetes patients. *P* < .05 was considered statistically significant.

Furthermore, we found that the absolute counts of neutrophils (4.1 [IQR, 2.8‐6.9] vs 2.5 [IQR, 1.6‐3.7]; Table 2), and the levels of CRP (32.8 [IQR, 11.3‐93] vs 16.3 [IQR, 7.17‐43.9]), ESR (67 [47.5‐81] vs 23 [10‐49]), as well as D‐dimer (1.15 [IQR, 0.83‐2.11] vs 0.54 [0.25‐1.1]) were significantly higher in diabetes group compared to non‐diabetes group. Beyond that, the absolute count of lymphocytes (0.86 [IQR, 0.5‐1.3] vs 0.97 [0.74‐1.3]) and red blood cells (3.9 [IQR, (3.5‐4.2)] vs 4.17 [3.8‐4.5]), and the level of haemoglobin (117 [IQR, 105‐123.5] vs 127 [117‐136]; Table 2) were significantly lower in diabetes group compared to non‐diabetes group. These data showed that the COVID‐19 patients with diabetes are at higher risk of excessive uncontrolled inflammation responses and hypercoagulable state, which may contribute to a poorer prognosis of COVID‐19.

Studies have shown that comorbidities, like chronic obstructive pulmonary disease, hypertension and malignancy, may predispose to poorer clinical outcomes.^13^ Therefore, we further explored the impact of comorbidities on the prognosis of the COVID‐19 patients without diabetes, and we found that levels of HBDH (208 [IQR, 147‐264.5] vs 141.5 [IQR, 124.75‐150.5]), ALT (27 [IQR, 19‐47] vs 18.5 [IQR, 13‐24]), LDH (281 [IQR, 196‐388] vs 186.5 [IQR, 177‐204.5]), γ‐glutamyltransferase (GGT; 28 [IQR, 17‐55] vs 13 [IQR, 11‐15.25]) and CRP (25.4 [IQR, 11.8‐54.7] vs 7.43 [IQR, 3.14‐13.45]), serum ferritin (434.5 [267.2‐710.4] vs 128.9 [57.25‐193.15]), ESR (26 [13‐58] vs 8 [7‐26]), IL‐6 (13.7 [IQR, 5.8‐28.2] vs 4.13 [IQR, 3.14‐10.61]), as well as D‐dimer (0.67 [IQR, 0.32‐1.25] vs 0.25 [0.22‐0.31]), FIB (4.79 [IQR, 3.8‐5.9] vs 3.75 [IQR, 3.04‐4.75]) and the absolute count of neutrophils (2.5 [IQR, 1.6‐4.2] vs 2.54 [IQR, 2.05‐3.22]) were significantly higher in comorbidity group compared to non‐comorbidity group. Beyond that, the absolute counts of lymphocytes (0.95 [IQR, 0.73‐1.2] vs 1.33 [1.17‐1.63]) and red blood cells (4.1 [IQR, (3.7‐4.4)] vs 4.36 [4.14‐4.64]), and the level of haemoglobin (125.5 [IQR, 115.8‐136] vs 133 [120‐137.75]; Table S1) were significantly lower in comorbidity group compared to non‐comorbidity group. These data showed that the COVID‐19 patients with comorbidity is at higher risk of tissue injury‐related enzymes release, excessive uncontrolled inflammation responses and hypercoagulable state, which may signifying a poorer prognosis of COVID‐19. These results prove that comorbidities do have an impact on the progression and prognosis of COVID‐19.

Since our purpose is to explore whether diabetes is a risk factor for the progression and prognosis of COVID‐19, we next excluded patients with comorbidities other than diabetes to avoid the impact of other comorbidities on the results. Our findings are as follows. Compared with patients without diabetes, patients with diabetes were older (61 [IQR, 57‐69] vs 32 [IQR, 30‐37]), had more nausea and vomiting (16.7% vs 0%) and higher mortality(16.7% vs 0%), but had no significant differences in gender and other baseline symptoms, follow‐up time, as well as the time from onset of symptom to hospital admission between the two groups (Table 3).

**TABLE 3:** Demographics and baseline characteristics of diabetic and non‐diabetic COVID‐19 patients without other comorbidities

	No. (%) Total (n = 50)	Non‐diabetes (n = 26)	Diabetes (n = 24)	*P*‐value^a^
Age, median (IQR), y	41 (32‐60)	32 (30‐37)	61 (57‐69)	<.01
Gender				
Male	21 (42)	9 (34.6)	12 (50)	.27
Female	29 (58)	17 (65.4)	12 (50)	
Signs and symptoms				
Fever	40 (80)	22 (84.6)	18 (75)	.30
Highest temperature, °C				
<37.3	9 (18)	4 (15.4)	5 (20.8)	.62
37.3 to 38.0	10 (20)	6 (23.1)	4 (16.7)	.57
38.1 to 39.0	26 (52)	15 (57.7)	11 (45.8)	.40
>39.0	4 (8)	1 (3.8)	3 (12.5)	.26
Fatigue	14 (28)	9 (34.6)	5 (20.8)	.27
Chill	39 (78)	20 (76.9)	19 (79.2)	.85
Cough	26 (52)	15 (57.7)	11 (45.8)	.84
Sputum production	12 (24)	7 (26.9)	5 (20.8)	.61
Pharyngalgia	4 (8)	4 (15.4)	0	.05
Dizziness	6 (12)	2 (7.7)	4 (16.7)	.33
Headache	4 (8)	3 (11.5)	1 (4.2)	.34
Chest tightness	6 (12)	4 (15.4)	2 (8.3)	.44
Chest pain	1 (2)	1 (3.8)	0	.33
Shortness of breath	9 (18)	4 (15.4)	5 (20.8)	.62
Myalgia	7 (14)	4 (15.4)	3 (12.5)	.77
Nausea and vomiting	4 (8)	0	4 (16.7)	.03
Diarrhoea	7 (14)	4 (15.4)	3 (12.5)	.29
Onset of symptom to, median (IQR), d				
Hospital admission	7 (5‐10)	7 (4.5‐10)	10 (6‐12)	.19
Mortality	4 (8)	0	4 (16.5)	.03

a *P* values indicate differences between diabetes and non‐diabetes patients. *P* < .05 was considered statistically significant.

**FIGURE 1:**
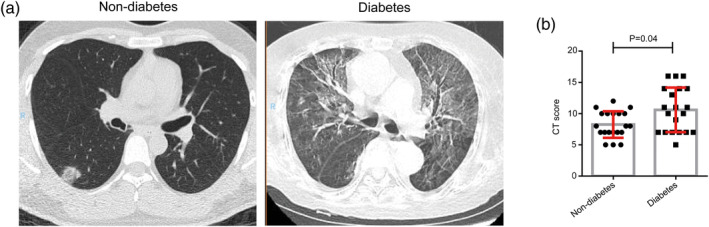
CT results of the patients with diabetes and patients without diabetes. A. The representative CT images of the patients with diabetes and patients without diabetes. B, The CT score of the patients with diabetes and patients without diabetes. *P* < .05 was considered statistically significant. CT, computed tomography

On admission, abnormalities in chest CT images were detected among all patients. The prominent radiologic abnormalities were bilateral ground‐glass opacity and subsegmental areas of consolidation, which is consistent with other recent reports.^1^ The representative chest CT imaging of patients with or without diabetes were compared, and the latter showed more severe pathological changes than the former (Figure 1A). Furthermore, the severity of pathological changes was evaluated by the quantifiable score system described before. We found that the diabetes group presented higher CT imaging score compared with non‐diabetes group (Figure 1B).

**FIGURE 2:**
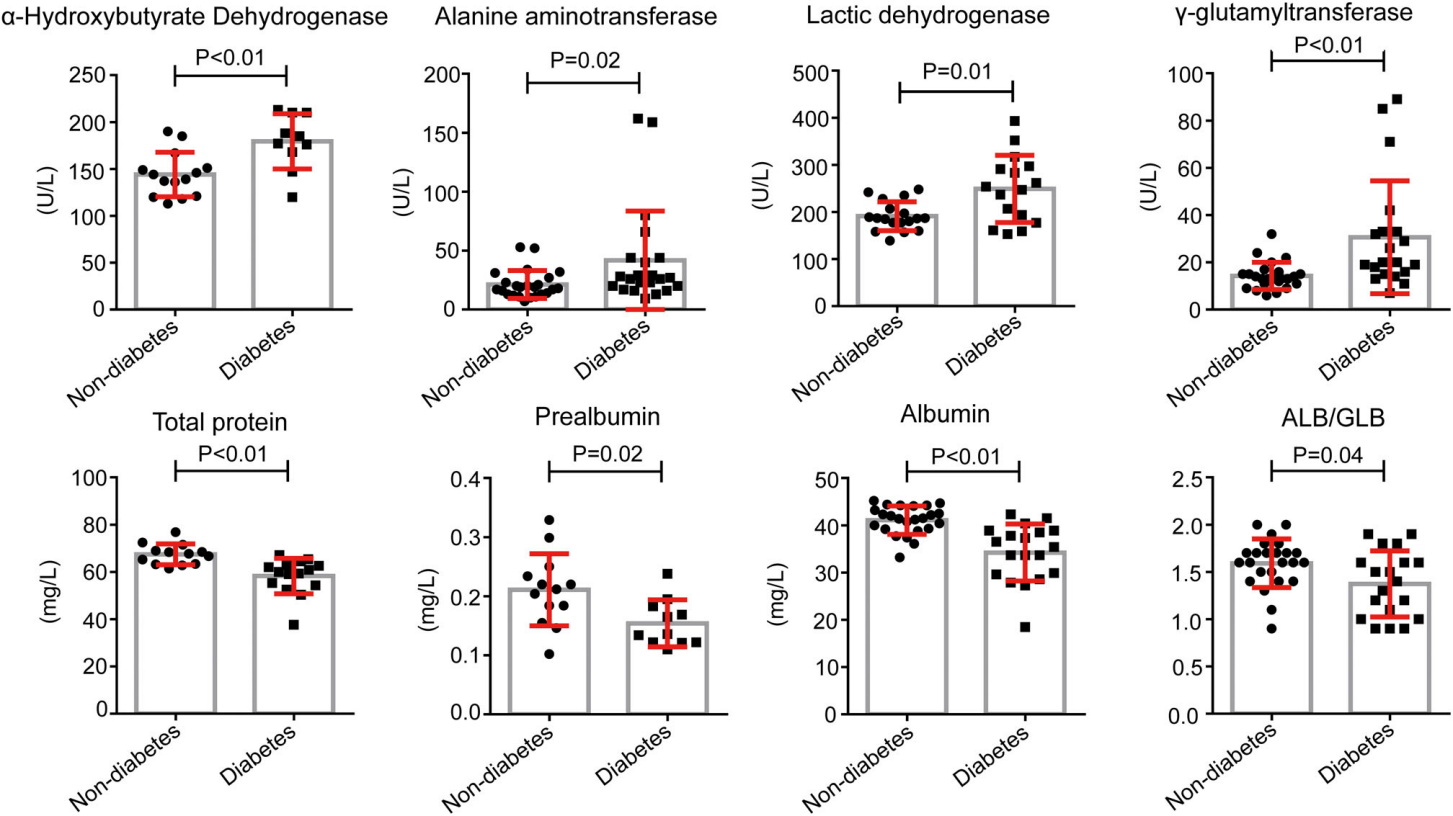
Biochemical examination results of the patients with diabetes and patients without diabetes. *P* < .05 was considered statistically significant

**FIGURE 3:**
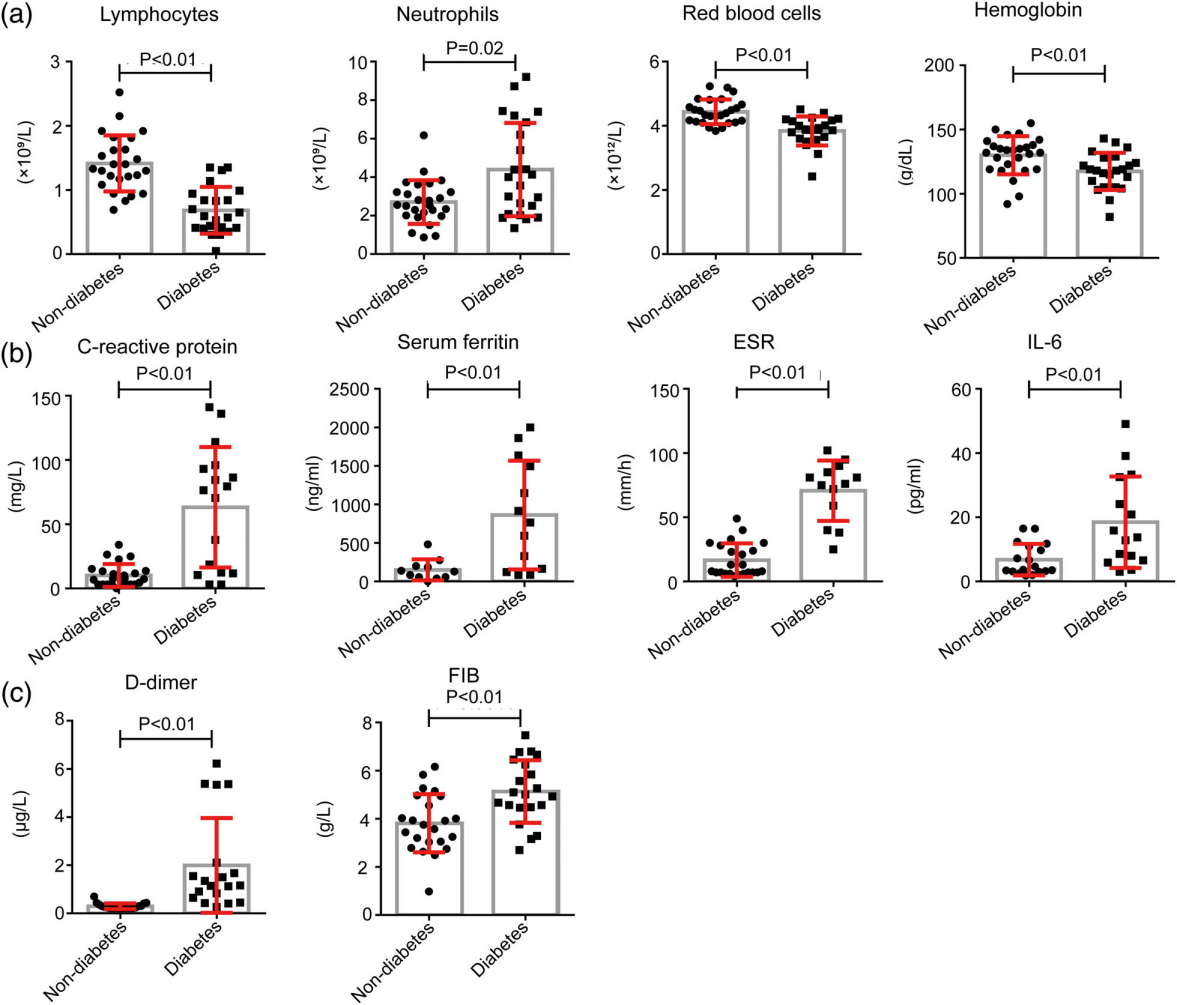
Other laboratory tests of the patients with diabetes and patients without diabetes. A, Blood test results of the patients with diabetes and patients without diabetes. B, Inflammation‐related laboratory results of the patients with diabetes and patients without diabetes. C, Coagulation‐related laboratory results of the patients with diabetes and patient without diabetes. *P* < .05 was considered statistically significant

It is worthy to note that levels of HBDH (181 [IQR, 170‐204.5] vs 141.5 [IQR, 124.75‐150.5]), ALT (24.5 [IQR, 20.25‐38.75] vs 18.5 [IQR, 13‐24]), LDH (250.5 [IQR, 189.6‐292.5] vs 186.5 [IQR, 177‐204.5]), GGT (20 [IQR, 15.75‐33] vs 13 [IQR, 11‐15.25]; Table 4 and Figure 2) and neutrophils (4 [IQR, 2.3‐6.52] vs 2.54 [IQR, 2.05‐3.22]; Table 4 and Figure 3), and CRP (76.4 [IQR, 12.4‐93] vs 7.43 [IQR, 3.14‐13.45]), serum ferritin (764.8 [164‐1496] vs 128.9 [57.25‐193.15]), ESR (76 [59‐85] vs 8 [7‐26]), IL‐6 (13.73 [IQR, (7.28‐28.31)] vs 4.13 [IQR, (3.14‐10.61)]), as well as D‐dimer (1.16 [IQR, 0.74‐1.89] vs 0.25 [0.22‐0.31]), FIB (5.01 [IQR, 4.48‐6.25] vs 3.75 [IQR, 3.04‐4.75]) were significantly higher in diabetes group compared to non‐diabetes group. Beyond that, the levels of total protein (60 [IQR, 54.8‐62.8] vs 67.7 [63.4‐69]), prealbumin (0.14 [IQR, 0.12‐0.18] vs 0.21 [0.18‐0.23]), albumin (35.4 [IQR, 29.75‐38.7] vs 41.45 [39.28‐43.43]) and ALB/GLB (1.4 [IQR, (1.05‐1.6)] vs 1.6 [1.48‐1.7]; Table 4 and Figure 2), as well as the absolute counts of lymphocytes (0.59 [IQR, 0.41‐0.89] vs 1.33 [1.17‐1.63]) and red blood cells (3.88 [IQR, (3.63‐4.16)] vs 4.36 [4.14‐4.64]), and the level of haemoglobin (118 [IQR, 107.5‐126] vs 133 [120‐137.75]; Table 4 and Figure 3) were significantly lower in diabetes group compared to non‐diabetes group. These data showed that diabetes may contribute to a poorer prognosis of COVID‐19.

**TABLE 4:** Comparison of laboratory parameters between diabetic and non‐diabetic COVID‐19 patients without other comorbidities

		Median (IQR)			
	Normal range	Total (n = 50)	Non‐diabetes (n = 26)	Diabetes (n = 24)	*P*‐value^a^
HBDH (U/L)	72 to 182	150 (136.75‐185)	141.5 (124.75‐150.5)	181 (170‐204.5)	<.01
ALT(U/L)	5 to 35	20.5 (16‐30.5)	18.5 (13‐24)	26.5 (20‐43)	.02
LDH (U/L)	109 to 245	195.4 (177‐247.75)	186.5 (177‐204.5)	250.5 (189.6‐292.5)	.01
GGT (U/L)	11 to 50	15 (13‐22.5)	13 (11‐15.25)	20 (15.75‐33)	<.01
Total protein (mg/L)	64 to 83	63.1 (59.83‐67.25)	67.7 (63.4‐69)	60 (54.8‐62.8)	<.01
Prealbumin (mg/L)	0.17 to 0.42	0.18 (0.14‐0.22)	0.21 (0.18‐0.23)	0.14 (0.12‐0.18)	.02
Albumin (mg/L)	35 to 55	39.2 (35.75‐42.1)	41.45 (39.28‐43.43)	35.4 (29.75‐38.7)	<.01
ALB/GLB	1.5 to 2.5	1.6 (1.3‐1.7)	1.6 (1.48‐1.7)	1.4 (1.05‐1.6)	.04
Lymphocytes (×10^9^/L)	1.1 to 3.2	1.04 (0.64‐1.36)	1.33 (1.17‐1.63)	0.59 (0.41‐0.89)	<.01
Neutrophils (×10^9^/L)	1.8 to 6.3	2.91 (2.09‐4.13)	2.54 (2.05‐3.22)	4 (2.3‐6.52)	.02
Red blood cells (×10^12^/L)	3.8 to 5.1	4.16 (3.88‐4.47)	4.36 (4.14‐4.64)	3.88 (3.63‐4.16)	<.01
Haemoglobin (g/dL)	115 to 150	124 (116‐135)	133 (120‐137.75)	118 (107.5‐126)	<.01
C‐reactive protein (mg/L)	<8	11.8 (3.14‐37.8)	7.43 (3.14‐13.45)	76.4 (12.4‐93)	<.01
Serum ferritin (ng/ml)	21.8 to 275	193.15 (85.73‐802.2)	128.9 (57.25‐193.15)	764.8 (164‐1496)	<.01
ESR (mm/h)	<15	26.5 (7‐62.25)	8 (7‐26)	76 (59‐85)	<.01
IL‐6 (pg/ml)	0.1 to 2.9	7.99 (3.52‐15.86)	4.13 (3.14‐10.61)	13.73 (7.28‐28.31)	<.01
D‐dimer (μg/L)	<0.5	0.42 (0.24‐1.15)	0.25 (0.22‐0.31)	1.16 (0.74‐1.89)	<.01
FIB (g/L)	2.0 to 4.0	4.52 (3.28‐5.27)	3.75 (3.04‐4.75)	5.01 (4.48‐6.25)	<.01

Abbreviations: ALB, albumin; ALT, alanine aminotransferase; COVID‐19，coronavirus disease 2019; ESR, erythrocyte sedimentation rate; FIB, fibrinogen; GGT, γ‐glutamyltransferase; GLB, globulin; HBDH, α‐hydroxybutyrate dehydrogenase; IQR, interquartile range; LDH, lactic dehydrogenase.

a *P* values indicate differences between diabetes and non‐diabetes patients. *P* < .05 was considered statistically significant.

Further, we analysed the effect of SARS‐CoV‐2 on the pathology of diabetes. We found that patients with diabetes control blood glucose levels with insulin or oral medicine before admission. Among them, 29.2% of the patients took insulin before and increased the dose of insulin after admission, and 37.5% of the patients took oral medicine before admission and started insulin therapy after admission, which meant that patients had poor glycemic control during hospitalization. Of all the diabetic patients, only three had diabetic complications, and those patients with diabetic complications were more likely to die as shown in Table 5.

**TABLE 5:** Treatments and complications of diabetic COVID‐19 patients without other comorbidities

	No. (%) Yes	No	*P*‐value^a^
Insulin therapy pre‐hospital	7 (29.2)	17 (70.8)	
Mortality	1	3	1
Insulin dose increased in hospital	7 (29.2)	17 (70.8)	
Mortality	1	3	1
Start insulin therapy after admission	9 (37.5)	15 (62.5)	
Mortality	3	1	.13
Diabetic complications	3 (12.5)	21 (87.5)	
Mortality	2 (66.7)	2 (9.5)	.045
Diabetic ketoacidosis	2 (12.5)	22 (87.5)	
Mortality	1 (50)	3 (13.6)	.31
Infectious shock	1 (4.2)	23 (95.8)	
Mortality	1 (100)	3 (13)	.17

a *P* values indicate differences between the two groups. *P* < .05 was considered statistically significant.

Finally, we explored the impact of comorbidities on the prognosis of patients with diabetes. Through our analysis, although the indicators for diabetic patients with other comorbidities are slightly higher than those without other comorbidities, no matter evaluated from organ damage, inflammatory factors or hypercoagulability, other comorbidities have little effect on the prognosis of patients with diabetes (Table S2).

## 4 DISCUSSION

Common perceptions associate diabetes with a generally increased mortality and morbidity to infectious diseases, although epidemiologic data that would prove this are surprisingly scarce. However, it seems to be confirmed that diabetes predisposes to certain types of infection and death,^6^, ^7^, ^8^, ^9^, ^11^ but it is still unknown whether diabetes is a risk factor for the prognosis of COVID‐19. Type 2 diabetes is widely viewed as a chronic, low‐grade inflammatory disease caused by long‐term immune system imbalance, metabolic syndrome or nutrient excess associated with obesity.^14^, ^15^ Obesity‐associated inflammation is characterized by an increased abundance and activation of innate and adaptive immunity cells in adipose tissue along with an increased release of inflammatory factors and chemokines locally and systemically.^16^ On the other side, data from human and animal studies suggest that some viruses are diabetogenic.^17^ Jali et al^18^ reported two individuals presenting with acute insulin dependent diabetes mellitus for a brief and transient period after being infected with chicken pox. And in a study of SARS, Yang et al^11^ found that even in non‐severe patients who have not been treated with glucocorticoid drugs, their fasting blood glucose levels are higher. Another study found that the immunostaining of ACE2 protein is strong in islets, but weak in exocrine tissues which means coronavirus might cause diabetes by damaging islets seriously.^19^ Since viral infection may cause sharp fluctuation of blood glucose level of diabetes patients, which adversely affect the recovery of patients, there is a reason to suspect that diabetes combined with SARS‐CoV‐2 pneumonia may form a vicious circle, which is detrimental to the prognosis of COVID‐19.

Reports showed that ICU patients, non‐ICU patients and recovery patients differ in CT imaging results, which means CT results can be used as one of the indicator for determining the severity of the SARS‐CoV‐2 pneumonia.^20^ According to the quantifiable score, we found that the diabetes group presented higher CT imaging score compared with non‐diabetes group, which means pneumonia in diabetic patients is more severe than non‐diabetic patients.

In addition to imaging results, laboratory results also give us some hints. Biochemical results showed that some indicative enzymes were abnormally elevated in the blood of patients with SARS‐CoV‐2 pneumonia, including LDH, HBDH, ALT and GGT, which indicated the injury of myocardium, kidney and liver. This result is consistent with the extensive distribution of SARS‐CoV‐2 receptors ACE2,^5^ and can also partially explain why some patients died from multiple organ failure.^1^ It is important to note that the levels of these enzymes were even higher in patients with diabetes when compared to patients without diabetes, which give us a clue that the injure of organs was much more serious in diabetes patients group than those without diabetes. In addition, our study found that the levels of total protein, albumin, prealbumin and haemoglobin are significantly lower in patients with diabetes compared to individuals without diabetes, which means diabetes patients are more likely to be undernourished.

According to a recent report, after analysing 138 hospitalized patients with COVID‐19, they found that neutrophilia related to cytokine storm induced by virus invasion, coagulation activation related to sustained inflammatory response and acute kidney injury related to direct effects of the virus might be associated with the death of patients with COVID‐19.^12^ And other study also found that patients with SARS‐CoV‐2 pneumonia, especially those with severe pneumonia, have significantly reduced lymphocyte counts and significantly increased inflammatory factors, such as IL‐6.^20^ In fact, in the advanced stage of SARS and Ebola virus infections, cytokine storms are also the main cause of eventual death for many patients. In our study, we found that, compared to patients without diabetes, absolute count of lymphocytes in peripheral blood of patients with diabetes is significantly lower, while the absolute count of neutrophils is remarkably higher. Furthermore, the serum levels of some inflammation‐related biomarker are much higher compared to those without diabetes, such as IL‐6, serum ferritin, ESR and CRP. It's noteworthy that for diseases that can induce a cytokine storm. IL‐6 is a very good predictor of disease severity and prognosis, and its expression time is longer than other cytokines (TNF and IL‐1).^21^ In addition, a significant rise in serum ferritin indicates the activation of the monocyte‐macrophage system, which is a crucial part of inflammatory storm. These results indicate that patients with diabetes are susceptible to form an inflammatory storm, which eventually lead to rapid deterioration of COVID‐19.

During the inflammatory storm, the D‐dimer increases significantly. In the early stage, this is the result of inflammation activating plasmin. However, as inflammation progresses and the presence of hypoxia, hypoxia‐induced molecules can activate thrombin directly, and the activation of monocyte‐macrophages would also secrete a mass of tissue factors and activate the exogenous coagulation pathway, which lead to an overall hypercoagulable state or even disseminated intravascular coagulation. In our study, we found that level of D‐dimer and FIB was significantly higher in patients with diabetes, which indicate that they are more prone to a hypercoagulable state than patients without diabetes.

Clinical medication showed that the insulin dose increased after the patient was infected with SARS‐CoV‐2, which shows that the virus has an impact on the patient's glucose metabolism. Dysregulation of glucose metabolism will aggravate diabetes and then affect the severity of pneumonia, which works as an amplification loop. Meanwhile, the diabetic complications signify the severity of diabetes, and these patients with diabetic complications showed a higher mortality rate, which further proves that diabetes is a risk factor for the prognosis of COVID‐19, and the severity of diabetes is positively correlated with the poor prognosis.

All in all, whether interference from other comorbidities is present or not, we found that SARS‐CoV‐2 pneumonia patients with diabetes are more severe than those without diabetes evaluating from organ damage, inflammatory factors or hypercoagulability, and are more likely to progress into a worse prognosis. Therefore, diabetes might could be considered as a risk factor for the outcome of SARS‐CoV‐2 pneumonia, and more intensive attention should be paid to patients with diabetes, in case of rapid deterioration.

## CONFLICT OF INTEREST

The authors declare that they do not have any conflict of interest regarding this publication.

## AUTHOR CONTRIBUTIONS

Desheng Hu and Shanshan Luo designed the study. Weina Guo, Mingyue Li, Yalan Dong, Haifeng Zhou, Zili Zhang, Chunxia Tian, and Renjie Qin researched data. Weina Guo and Mingyue Li contributed to the data analysis. Weina Guo, Mingyue Li, Yalan Dong, Keye Du, Haifeng Zhou, and Zili Zhang contributed to the discussion. Weina Guo and Mingyue Li wrote the manuscript. Haijun Wang, Yin Shen, Lei Zhao, Heng Fan, Shanshan Luo, and Desheng Hu reviewed/edited the manuscript.

## Supporting information


**Table S1** Comparison of laboratory parameters between non‐diabetic COVID‐19 patients with comorbidities and without comorbidities

**Table S2** Comparison of laboratory parameters between diabetic COVID‐19 patients with and without other comorbidities
