# Supplementary material for: Diabetes is a risk factor for the progression and prognosis of COVID‐19
Source: Diabetes Metab Res Rev. 2020 Apr 7;36(7):e3319. doi: 10.1002/dmrr.3319 (PMC7228407; doi:10.1002/dmrr.3319)
Supplement: Supplementary file 1 — Table S1 Comparison of laboratory parameters between non‐diabetic COVID‐19 patients with comorbidities and without comorbidities [file DMRR-36-0-s001.docx]

**Table S1. Comparison of laboratory parameters between non-diabetic COVID-19 patients with comorbidities and without comorbidities**

|  |  | Median (IQR) |  |  |  |
| --- | --- | --- | --- | --- | --- |
|  | **Normal Range** | **Total**  **(n=137)** | **Non-comorbidity (n=26)** | **Comorbidity**  **(n=111)** | ***P* Value^a^** |
| HBDH (U/L) | 72-182 | 190  (143.5-251.5) | 141.5  (124.75-150.5) | 208  (147-264.5) | 0.01 |
| ALT(U/L) | 5-35 | 25(17-42) | 18.5 (13-24) | 27(19-47) | <0.01 |
| LDH (U/L) | 109-245 | 241  (187-372.3) | 186.5  (177-204.5) | 281  (196-388) | <0.01 |
| GGT (U/L) | 11-50 | 24(14-45) | 13 (11-15.25) | 28(17-55) | <0.01 |
| Lymphocytes (×10^9^/L) | 1.1-3.2 | 0.97(0.74-1.3) | 1.33 (1.17-1.63) | 0.95(0.73-1.2) | <0.01 |
| Neutrophils (×10^9^/L) | 1.8-6.3 | 2.5(1.6-3.7) | 2.54 (2.05-3.22) | 2.5(1.6-4.2) | <0.05 |
| Red blood cells (×10^12^/L) | 3.8-5.1 | 4.17(3.8-4.5) | 4.36 (4.14-4.64) | 4.1 (3.7-4.4) | <0.01 |
| Hemoglobin (g/dL) | 115-150 | 127  (117-136) | 133  (120-137.75) | 125.5  (115.8-136) | 0.2 |
| C-reactive protein (mg/L) | <8 | 20.25  (10.7-50) | 7.43  (3.14-13.45) | 25.4  (11.8-54.7) | <0.01 |
| Serum ferritin (ng/ml) | 21.8-275 | 372.6  (185.8-685.8) | 128.9  (57.25-193.15) | 434.5  (267.2-710.4) | <0.01 |
| ESR (mm/h) | <15 | 23(10-49) | 8 (7-26) | 26(13-58) | <0.01 |
| IL-6 (pg/ml) | 0.1-2.9 | 11.16(4.5-25) | 4.13 (3.14-10.61) | 13.7(5.8-28.2) | <0.01 |
| D-dimer (µg/L) | <0.5 | 0.54  (0.25-1.1) | 0.25  (0.22-0.31) | 0.67  (0.32-1.25) | <0.01 |
| FIB (g/L) | 2.0-4.0 | 4.58(3.7-5.6) | 3.75 (3.04-4.75) | 4.79(3.8-5.9) | <0.01 |

Abbreviation: IQR, interquartile range; COVID-19，coronavirus disease 2019； HBDH, α-Hydroxybutyrate Dehydrogenase; ALT, Alanine aminotransferase; LDH, Lactic dehydrogenase; GGT, γ-glutamyltransferase; ESR, erythrocyte sedimentation rate; FIB, fibrinogen. ^a^ *P* values indicate differences between diabetes and non-diabetes patients. *P* <0.05 was considered statistically significant.
