# Supplementary material for: Diabetes is a risk factor for the progression and prognosis of COVID‐19
Source: Diabetes Metab Res Rev. 2020 Apr 7;36(7):e3319. doi: 10.1002/dmrr.3319 (PMC7228407; doi:10.1002/dmrr.3319)
Supplement: Supplementary file 2 — Table S2 Comparison of laboratory parameters between diabetic COVID‐19 patients with and without other comorbidities [file DMRR-36-0-s002.docx]

**Table S2. Comparison of laboratory parameters between diabetic COVID-19 patients with and without other comorbidities**

|  |  | Median (IQR) |  | |  |  |
| --- | --- | --- | --- | --- | --- | --- |
|  | **Normal Range** | **Total**  **(n=37)** | **Non-comorbidity (n=24)** | | **Comorbidity**  **(n=13)** | ***P* Value^a^** |
| HBDH (U/L) | 72-182 | 210  (177-480) | 199  (176.3-318.8) | 346.5  (186-492) | | 0.8 |
| ALT(U/L) | 5-35 | 28 (21-34) | 26.5 (20-43) | 29 (20-40) | | 0.98 |
| LDH (U/L) | 109-245 | 252  (174.5-292.5) | 250.5  (189.6-292.5) | 254.5  (187.8-302) | | 0.65 |
| GGT (U/L) | 11-50 | 32  (17.5-52) | 20  (15.75-33) | 32  (17.5-53.5) | | 0.19 |
| Lymphocytes (×10^9^/L) | 1.1-3.2 | 0.86  (0.5-1.3) | 0.59  (0.41-0.89) | 1.25(0.82-1.44)  (0.44-1.26) | | 0.42 |
| Neutrophils (×10^9^/L) | 1.8-6.3 | 4.1  (2.8-6.9) | 4  (2.3-6.52) | 4.39  (2.6-6.9) | | 0.71 |
| Red blood cells (×10^12^/L) | 3.8-5.1 | 3.9  (3.5-4.2) | 3.88  (3.63-4.16) | 3.9  (3.6-4.2) | | 0.13 |
| Hemoglobin (g/dL) | 115-150 | 117  (105-123.5) | 118  (107.5-126) | 115  (105-122) | | 0.12 |
| C-reactive protein (mg/L) | <8 | 32.8  (11.3-93) | 76.4  (12.4-93) | 24.26  (5.8-64.3) | | 0.31 |
| Serum ferritin (ng/ml) | 21.8-275 | 594.4  (164-1146.2) | 764.8  (164-1496) | 329.4  (143.2-839.6) | | 0.15 |
| ESR (mm/h) | <15 | 67(47.5-81) | 76 (59-85) | 67(43.8-79.8) | | 0.98 |
| IL-6 (pg/ml) | 0.1-2.9 | 18.3  (7.3-37.6) | 13.73  (7.28-28.31) | 18.35  (7.3-34.7) | | 0.86 |
| D-dimer (µg/L) | <0.5 | 1.15  (0.83-2.11) | 1.16  (0.74-1.89) | 1.03  (1.89-1.51) | | 0.8 |
| FIB (g/L) | 2.0-4.0 | 5.1(4.6-6.3) | 5.01 (4.48-6.25) | 5.1(5-6.3) | | 0.02 |

Abbreviation: IQR, interquartile range; COVID-19，coronavirus disease 2019； HBDH, α-Hydroxybutyrate Dehydrogenase; ALT, Alanine aminotransferase; LDH, Lactic dehydrogenase; GGT, γ-glutamyltransferase; ESR, erythrocyte sedimentation rate; FIB, fibrinogen. ^a^ *P* values indicate differences between diabetes and non-diabetes patients. *P* <0.05 was considered statistically significant.
